# A new national survey of centers for cognitive disorders and dementias in Italy

**DOI:** 10.1007/s10072-023-06958-8

**Published:** 2023-08-18

**Authors:** Ilaria Bacigalupo, Francesco Giaquinto, Emanuela Salvi, Giulia Carnevale, Roberta Vaccaro, Fabio Matascioli, Giulia Remoli, Nicola Vanacore, Patrizia Lorenzini, the Permanent Table of the National Dementia Plan Study Group and the CCDDs Study Group, Gennarina Arabia, Alessandro Amorosi, Ilaria Bacigalupo, Anna Maria Bargagli, Luisa Bartorelli, Cristina Basso, Manuela Berardinelli, Maria Pompea Bernardi, Caterina B. N. A Bianchi, Lorenzo Blandi, Federica Boschi, Amalia Cecilia Bruni, Alessandra Caci, Paolo Caffarra, Marco Canevelli, Andrea Capasso, Susanna Cipollari, Mariapia Cozzari, Alfonso Di Costanzo, Teresa Di Fiandra, Annalisa Di Palma, Andrea Fabbo, Federica Francescone, Carlo Gabelli, Sabina Gainotti, Francesca Galeotti, Giuseppe Gambina, Marina Gasparini, Maria Assunta Giannini, Micaela Gilli, Marcello Giordano, Annarita Greco, Antonio Guaita, Fabio Izzicupo, Fiammetta Landoni, Elisa Lidonnici, Nicoletta Locuratolo, Giancarlo Logroscino, Alessandra Lombardi, Gilda Losito, Francesca Lubian, Maria Cristina Lupinetti, Sara Madrigali, Camillo Marra, Filippo Masera, Massimiliano Massaia, Antonio Mastromattei, Antonio Matera, Manlio Matera, Francesco Mazzoleni, Carla Melani, Serena Meloni, Elena Memeo, Marco Musso, Antonella Notarelli, Marco Onofrj, Ernesto Palummeri, Valeria Panetta, Carlo Petrini, Tommaso Piccoli, Alessandro Pirani, Stefano Piras, Gabriella Porro, Mario Possenti, Elena Rendina, Antonino Riolo, Luciana Riva, Emanuela Salvi, Sara Santini, Silvia Scalmana, Nando Scarpelli, Piero Secreto, Monica Seganfreddo, Stefano Sensi, Carla Severino, Patrizia Spadin, Patrizia Spallino, Anna Laura Spinelli, Andrea Stracciari, Marco Trabucchi, Nicola Vanacore, Antonio Zaccardi, Egidio Accardo, Omar Ahmad, Domenico Ajena, Giovanni Alba, Alberto Albanese, Andrea Albergati, Maria Alessandria, Pasquale Alfieri, Mario Alimenti, Angelo Aliprandi, Roberto Altavilla, Salvatore Amarù, Immacolata Ambrosino, Felice Amideo, Stefania Ammendola, Francesco Amoruso, Candida Andreati, Vincenzo Andreone, Rossano Angeloni, Francesco Annunziata, Sara Antenucci, Ildebrando Appollonio, Gennarina Arabia, Luciano Arcudi, Marianna Ardillo, Maria Carmela Gabriella Arena, Andrea Arighi, Gennaro Arpino, Anna Bagalà, Antonio Baiano, Antonio Balestrino, Mario Barbagallo, Marianna Barbuto, Cesare Bargnani, Paolo Barone, Antonella Bartoli, Claudia Bauco, Giuseppe Bellelli, Marco Antonio Bellini, Aldo Bellora, Giuseppe Benati, Sandro Beretta, Lucia Bergamini, Eleonora Bergonzini, Valentina Bessi, Angelo Bianchetti, Erika Bisio, Roberta Boiardi, Elisabetta Bollani, Laura Bologna, Francesco Bolzetta, Stefano Boni, Tiziano Borgogni, Gabriella Bottini, Ida Bottone, Angela Bove, Bossio Roberto Bruno, Giuseppe Bruno, Patrizia Bruno, Carmela Bucca, Manuela Buganza, Graziano Buzzi, Paolo Buzzi, Gabriella Cacchio’, Arturo Cafarelli, Viviana Cafazzo, Marcella Caggiula, Annachiara Cagnin, Gianluigi Calabrese, Giusi Alessandro Calabrese, Maria Calandra, Veronica Caleri, Donatella Calvani, Massimo Camerlingo, Roberto Cantello, Andrea Capasso, Sabina Capellari, Giovanni Capobianco, Maria Carmela Capoluongo, Rossana Cappelletti, Claudio Capra, Natalia Caravona, Carlo Maria Stucchi, Maria Alessandra Carluccio, Severina Carteri, Anna Casanova, Francescosaverio Caserta, Paolo Caso, Gaetana Cassaniti, Emanuele Cassetta, Silvia Casson, Vincenzo Castiello, Tatiana Cattaruzza, Anna Ceccon, Moira Ceci, Sabatino Cella, Silvia Cenciarelli, Bruno Censori, Giuliano Cerqua, Paolo Cerrone, Pasquale Cervera, Silvia Chemotti, Annalisa Chiari, Roberta Chiloiro, Luisa Cirilli, Raffaella Clerici, Alessandra Coin, Gianfranco Colacino, Francesco Paolo Colacioppo, Rosanna Colao, Antonio Colin, Brigida Coluccia, Giancarlo Maria Conti, Filomena Coppola, Francesca Coppola, Massimo Corbo, Antonello Cossu, Alfredo Costa, Gabriella Costa, Manuela Costa, Maria Sofia Cotelli, Salvatore Cottone, Maria Immacolata Cozzolino, Andrea Crucitti, Eduardo Cumbo, Antonio Currà, Carlo Dallocchio, Ferdinando D’amico, Anna D’Amore, Stefano De Carolis, Maurizio De Donato, Paola De Feo, Franz De La Pierre, Maria De Laurentiis, Ida De Lauretis, Gian Placido De Luca, Alessandro De Palma, Laura De Togni, Antonio Demontis, Dora D’Epiro, Giovambattista Desideri, Miranda Desiderio, Marco Di Donato, Gabriella Di Emidio, Raffaella Di Giacopo, Vincenzo Di Lazzaro, Rita Di Leo, Salvatore Di Marco, Gaetano Di Quarto, Babette Dijk, Natasa Dikova, Maria Stefania Dioguardi, Federica Dominici, Michele Dotta, Carla Dotti, Domenica Esposito, Sabrina Esposito, Zaira Esposito, Evaristo Ettorre, Andrea Fabbo, Giovanna Faccenda, Angelamaria Falanga, Michela Falorni, Fraia Falvo, Agostina Fappani, Farina Elisabetta Ismilde Mariagiovanna, Sara Fascendini, Francesco Fattapposta, Irene Favatella, Grazia Daniela Femminella, Salvatore Ferrara, Patrizia Ferrari, Alessandra Ferraris, Franco Ferraro, Raffaele Ferri, Salvatore Ferrigno, Francesco Filastro, Massimo Filippi, Antonio Finelli, Chiara Finelli, Maria Rita Fiori, Francesco Fiorillo, Gianluca Floris, Anna Fontanella, Luigi Forgione, Andrea Foti, Francesca Fulvia Foti, Neviani Francesca, Fabio Frediani, Giovanni Frontera, Maria Luigia Fulgido, Carmine Fuschillo, Luciano Gabbani, Carlo Gabelli, Franco Galati, Renato Galli, Angelo Gallo, Livia Gallo, Maurizio Gallucci, Gabriella Galluccio, Pietro Gareri, Lorenzo Gasperi, Giovanni Gelmini, Michele Gennuso, Carmela Gerace, Daria Ghersetti, Federica Giambattistelli, Valter Giantin, Bernardo Giordano, Maurizio Giorelli, Agata Giorgianni, Franco Giubilei, Laura Godi, Luciano Gorelli, Daniela Gragnaniello, Serena Granziera, Giuseppe Greco, Rodolfo Grella, Michele Grieco, Luigi Grimaldi, Maria Guarino, Chiara Guarnerio, Giovanni Guidi, Leonello Guidi, Lucia Iallonardo, Alessandro Iavarone, Tiziana Ingegni, Pasqualina Insardà, Claudio Ivaldi, Fabio Izzicupo, Carmelo Roberto Labate, Roberto Lacava, Francesco Lalli, Anna Maria Lammardo, Paolo Massimo Laurienzo, Alessandro Leonardi, Maria Rosa Leotta, Rosario Leuzzi, Simona Linarello, Pasqualino Litterio, Daniele Lo Coco, Mario Rosario Lo Storto, Chiara Logi, Francesco Ottavio Logullo, Alessandra Lombardi, Fortunato Lombardi, Antonio Lorido, Francesco Antonio Losavio, Francesca Lubian, Antonina Luca, Massimo Lenzi Lucio, Livia Ludovico, Maria Lunardelli, Mariarosaria Lupo, Simona Luzzi, Maurizio Maddestra, Gennaro Maio, Mariangela Maiotti, Anna Maria Malagnino, Giovanni Mancini, Angela Manica, Michele Maniscalco, Barbara Manni, Antonio Manucra, Laura Manzoni, Marco Marabotto, Giuseppe Marchesiello, Michela Marcon, Alessandra Marcone, Roberto Marconi, Alessandro Margiotta, Angela Marianantoni, Donatella Mariani, Gemma Marino, Saverio Marino, Vito Marinoni, Angela Marra, Camillo Marra, Maria Marrari, Mabel Martelli, Alessandro Marti, Alessandro Martorana, Martina Marvardi, Saverio Mascolo, Massimiliano Massaia, Vita Maria Alba Mastronuzzi, Maria Letizia Mazzi, Andrea Mazzone, Rossella Mecacci, Patrizia Mecocci, Deidania Medici, Daniele Mei, Gian Giuseppe Melandri, Maurizio Melis, Francesca Meneghello, Vanda Menon, Carmen Menza, Paola Merlo, Graziella Milan, Antonio Milia, Calogero Claudio Millia, Sergio Minervini, Carolina Anna Mobilia, Massimo Moleri, Elena Molteni, Giovanni Moniello, Stefano Montanari, Maria Teresa Mormile, Giuseppe Moro, Gianluca Moscato, Enrico Mossello, Angela Domenica Mundo, Giuseppe Mura, Fabio Musca, Anna Maria Musso, Anna Nardelli, Viviana Nicosia, Vincenzo Nociti, Alessio Novelli, Francesco Nuccetelli, Marco Onofrj, Lorenza Orefice, Daniele Orsucci, Alfonso Pace, Cristina Paci, Roberta Padoan, Alessandro Padovani, Lorenzo Palleschi, Maria Teresa Palmisani, Marco Palmucci, Pasquale Palumbo, Nadia Rita Panico, Antonella Pansini, Roberta Pantieri, Paolo Paolello, Salotti Paolo, Matteo Pardini, Lucilla Parnetti, Emma Parrotta, Michela Passamonte, Agostino Pastore, Ebe Pastorello, Luca Pelini, Morena Pellati, Mario Pellegrino, Giuseppe Pelliccioni, Maria Giovanna Pennisi, Michele Perini, Daniele Perotta, Diego Persico, Virginia Petrella, Fabia Petri, Maristella Piccininni, Laura Pierguidi, Antonella Pierobon, Alessio Pietrella, Alberto Pilotto, Patrizia Pinto, Alessandro Pirani, Vincenzo Pizza, Domenico Plantone, Massimiliano Plastino, Patrizia Poddighe, Simone Pomati, Angela Pompilio, Marialuisa Pontecorvo, Alessandro Prelle, Giorgio Previderè, Ennio Pucci, Gianfrano Puoti, Valeria Putzu, Annaflavia Rabasca, Massimo Raffaele, Innocenzo Rainero, Claudia Rais, Michele Rana, Alberto Ranzenigo, Giovanni Rea, Enrico Righetti, Giuseppe Rinaldi, Augusto Rini, Maria Rosaria Rizzo, Massimo Rizzo, Paola Rocca, Laura Roffredo, Daniela Roglia, Franco Romagnoni, Carlo Romano, Annalisa Romasco, Leonardo Romeo, Stefano Ronzoni, Chiara Emilia Rosci, Mara Rosso, Renzo Rozzini, Eleonora Ruberto, Stefania Ruberto, Gregorio Rungger, Giovanni Ruotolo, Francesco Russo, Giuseppe Russo, Roncacci Sabina, Simona Sacco, Giorgio Sacilotto, Giuseppe Salemi, Paolo Salotti, Elena Salvatore, Luisa Sambati, Giuseppe Sanges, Francesco Santamaria, Ignazio Michele Santilli, Mariangela Santoro, Riccardo Saponara, Monica Scarmagnan, Fabrizio Scataglini, Loredana Seccia, Vladimir Selmo, Stefano Sensi, Luigi Sicurella, Antonello Silvestri, Massimo Simone, Antonella Sirca, Intissar Sleiman, Paolo Solla, Gianfranco Spalletta, Sarah Anna Sperber, Laura Spinelli, Franz Spoegler, Patrizia Sucapane, Domenico Suraci, Benedetta Tagliabue, Stefania Tagliente, Elena Tamietti, Gianluca Tedeschi, Antonio Tetto, Alessandro Tiezzi, Pietro Tiraboschi, Gloria Tognoni, Carmine Tomasetti, Francesco Torchia, Giuseppe Toriello, Giovanna Trevisi, Gabriele Tripi, Giuseppe Trombetta, Alessandro Tulliani, Antonella Rita Vaccina, Luca Valentinis, Gina Varricchio, Giuliano Antonella Vasquez, Filomena Vella, Federico Verde, Chiara Verlato, Giuliana Vezzadini, Simone Vidale, Assunta Vignoli, Daniele Villani, Alfredo Vitelli, Luigina Volpentesta, Gino Volpi, Domenico Vozza, Patrizia Wanderlingh, Christian Wenter, Davide Zaccherini, Massimo Zanardo, Giampietro Zanette, Michela Zanetti, Orazio Zanetti, Carla Zanferrari, Marta Zuffi, Vincenzo Zupo

**Affiliations:** 1 National Centre for Disease Prevention and Health Promotion, Italian National Institute of Health, Via Giano Della Bella 34, 00161 Rome, Italy; 2 Department of Human and Social Sciences, University of Salento, Lecce, Italy; 3 National Center for Drug Research and Evaluation, Italian National Institute of Health, Rome, Italy; 4 Italian National Institute of Health FONDEM Study Group, Rome, Italy; 5 GINCO, Aware Aging Group, Como, Italy; 6 TAM Onlus, Social Cooperative, Naples, Italy; 7 Neurology Section, University of Milano-Bicocca, Milan, Italy

**Keywords:** Memory clinic, Centers for Cognitive Disorders and Dementias, Survey, COVID-19, Dementia, Public health, National dementia plan

## Abstract

**Introduction:**

A new national survey has been carried out by the Italian Centers for Cognitive Disorders and Dementias (CCDDs). The aim of this new national survey is to provide a comprehensive description of the characteristics, organizational aspects of the CCDDs, and experiences during the COVID-19 pandemic.

**Methods:**

A list of all national CCDDs was requested from the delegates of each Italian region. The online questionnaire is divided in two main sections: a profile section, containing information on location and accessibility, and a data collection form covering organization, services, treatments, activities, and any service interruptions caused by the COVID-19 outbreak.

**Results:**

In total, 511 out of 534 (96%) facilities completed the profile section, while 450 out of 534 (84%) CCDDs also completed the data collection form. Almost half of the CCDDs (55.1%) operated for 3 or fewer days a week. About one-third of the facilities had at least two professional figures among neurologists, geriatricians and psychiatrists. In 2020, only a third of facilities were open all the time, but in 2021, two-thirds of the facilities were open.

**Conclusion:**

This paper provides an update on the current status of CCDDs in Italy, which still shows considerable heterogeneity. The survey revealed a modest improvement in the functioning of CCDDs, although substantial efforts are still required to ensure the diagnosis and care of patients with dementia.

**Supplementary Information:**

The online version contains supplementary material available at 10.1007/s10072-023-06958-8.

## Introduction

The World Health Organization (WHO) has recognized dementia as a priority in public health and has endorsed the Global Action Plan on the public health response to dementia 2017–2025 [1]. In Italy, about one million individuals are affected by dementia and around 900,000 by mild cognitive impairment (MCI), with more than three million people directly or indirectly involved in caring [2, 3]. Recent population-based studies conducted worldwide have suggested that the age-specific risk of dementia may be changing in some geographical areas [4]. Therefore, in Italy, the number of cases is increasing due to aging population, and current projections estimate that by 2025 there will be 1.5 million people living with dementia in Italy [5]. However, by modifying 12 risk factors it may be possible to prevent or delay up to 40% of dementia cases [6].

Memory clinics are healthcare services that have a pivotal role in the management of dementia and cognitive disturbance [7].

An emerging body of evidence shows that timely access to a specialist cognitive assessment service and early diagnosis lead to better outcomes for people with dementia and their families, and are cost-effective for the health system [8]. Early intervention helps to delay cognitive decline, preserve functional abilities, and delay admission to institutional care [9]. Accurate and timely diagnosis, support for persons living with dementia and their families, and early intervention provided by a multidisciplinary team of health professionals, together with legal advice, can have a positive impact on care [7].

However, a significant heterogeneity in the structure, organization, resources, and activities of memory clinics has been documented in several countries, particularly those that are included in the Organization for Economic Co-operation and Development (OECD) [10–16], potentially attributable to the absence of care guidelines and standards [10].

There are currently no disease-modifying therapies for Alzheimer’s disease (AD) in Europe. However, new drugs will be approved in Europe in the next years [17], while the composition of the dementia care workforce continues to evolve [18].

A substantial number of MCI cases would require screening, diagnosis, and treatment to prevent the progression of Alzheimer’s dementia [19]. Therefore, it is crucial to assess the preparedness of memory clinics before the introduction of new therapies. Therefore, the public health system must ensure that the resources are in place to provide adequate care. Unfortunately, there are numerous barriers to achieving this goal, and understanding the location, accessibility, organization, services, and treatments provided by memory clinics is a prerequisite for resource allocation.

Moreover, during the COVID-19 pandemic, many patients experienced difficulties in accessing care with delays in diagnosis and extended follow-up periods [20, 21].

The Italian memory clinics, initially established as Alzheimer’s Evaluation Units, were introduced in 2000 with the Cronos project and [22] were renamed as Centers for Cognitive Disorders and Dementias (CCDDs) with the formulation of the Italian National Dementia Plan (NDP) in October 2014 [23]. The CCDDs are public services fully covered by the national health system [23], which are important to ensure timely recognition and diagnosis of cognitive disorders. Furthermore, they are responsible for prescribing anti-dementia drugs [24] and antipsychotics drugs in patients with dementia [24] and actively provide psychosocial, educational, and rehabilitative interventions and post-diagnosis psychosocial support for patients and caregivers.

In December 2020, the Italian Parliament finally approved an amendment to the 2021 budget law, which provided total funding of €15 million for the Italian National Dementia Plan from 2021 to 2023. The main goal of the Italian Fund for Alzheimer’s and other dementias is to provide new strategies from a public health perspective and to understand the management of dementia care in Italy [25]. This project has funded a new survey of the CCDDs located in Italy, and an update of the online map of these services [25].

A previous national survey of CCDDs was conducted in 2014, and provided a snapshot of the organization and activities of CCDDs in Italy [26–28]. In addition, a specific survey was conducted between December 2020 and April 2021 to collect data on the readiness of CCDDs to support the inclusion of migrants in the public health response to dementia [29].

This new national survey aims to illustrate the structure of Italian CCDDs, such as references, service and staff organization, diagnostic work-up, and experience during the pandemic period.

## Methods

### Surveyed facilities

In continuity with previous research [27], this study used a survey research approach. All CCDDs operating in Italy were included in the study. These facilities are located in different geographical area of the country. In order to promote coordinated action with the different regional and local health authorities, representatives of the Regions and Autonomous Provinces in the Permanent Table of the National Dementia Plan actively participated in compiling updated lists and contact details of CCDDs within their respective territories. Geographical macro areas were defined according to the categorization of the Italian National Institute of Statistics (ISTAT).

### Questionnaire

The survey questionnaire was based on the previous questionnaire to ensure the comparability of results [27] and was partially modified by members of the Permanent Table of the National Dementia Plan. It consists of two sections: a profile section and a data collection form.

The profile section (19 questions) gathered information regarding: (i) the address and contact details of the main facility and any branches; (ii) the setting of facility (e.g., territorial, hospital, university); (iii) the days and hours of operation on a weekly basis (both for the main facility and branches); and (iv) other information on services such as patients access, activation data, and the facility’s clinical director. Information was requested for the year 2022. Much of this information is used to update the Dementia Observatory website (https://www.demenze.it/ ).

The data collection form (20 questions) requires information about: (i) the staff composition; (ii) the availability of an integrated care pathway document and a computerized archive; (iii) the clinical and cognitive assessment tools used; (iv) the clinical activity (e.g., waiting list and the average waiting time for accessing to the service); (v) the number of patients evaluated annually and the type of diagnosis; and (vi) psychosocial, educational, and rehabilitation services provided directly or by agreements. It was requested to provide information for the year 2019, the last year before the pandemic.

Compared to the previous questionnaire [27], the presence of CCDDs with branches was investigated and structural information (number of locations, days and hours of operation) as well as contact information was collect for the inclusion in the online map. Information was collected on the presence of professionals not previously considered (e.g., occupational therapists, cultural mediators, and interpreters), the provision of telemedicine services and the use of digital monitoring tools. Finally, respondents were asked about the CCDDs activity during the pandemic years 2020 and 2021, whether the service was always open or partially closed, and the average length of any possible closure. The survey questionnaire was developed online by computer experts on a platform that allowed for continuous monitoring of the survey. The questionnaire included both closed questions with pre-coded response options and open-ended questions. Most questions were mandatory, and some automatic checks were implemented in the questionnaire to prevent inconsistent responses (e.g., the sum of the entered percentages should be 100).

### Procedure

Ten CCDDs participated in a pilot phase to test the system’s functionality. Then, the survey was launched in July 2022 and closed in February 2023. CCDDs were invited to participate in the study through a cover letter sent by e-mail. They were informed about who was responsible for the research, the aims of the study, and the objectives of the “Italian Fund for Alzheimer’s and other dementias”. They were also invited to participate in the online survey and received personal access credentials and a guide to facilitate data entry. They had the option to complete the survey in multiple sessions and it typically took between 30 and 60 min to complete. Clinical representatives of the services were responsible for completing the self-administered computer-based questionnaire. A team of researchers monitored survey participation and followed up with emails and phone calls to clarify any doubts about the survey. The representatives of the Regions and Autonomous Provinces in the Permanent National Table on Dementia actively collaborated with the National Institute of Health in involving the CCDDs in the study. After consent was obtained responses were automatically registered in the online platform and then exported for statistical analysis in accordance with the privacy policy.

### Statistical analysis

Before starting the analytical phase, a number of cross checks were conducted to ensure the consistency of the collected information, particularly regarding the number of patients under care and the date of service activation.

The mean number of dementia cases per CCDD was calculated by dividing the estimated cases of dementia by the total number of CCDDs in each Region or Autonomous Province. The cases of dementia in a specific Region or Autonomous Province were estimated by multiplying the European dementia prevalence rates stratified by age and gender [2] for the number of over-65-year residents in each Italian Region in 2022, as provided by the Italian National Institute of Statistics (http://dati.istat.it/).

Categorical variables were reported as absolute numbers and percentages; continuous parameters were described as median and interquartile range (IQR) and mean (min–max). The normality of the distribution of continuous variables was checked by the Shapiro Wilk test. The collected information characterizing the facilities was compared among the three Italian macro areas (North, Center, and South/Islands) using the Kruskal–Wallis test for continuous variables and Chi-square test or Fisher’s exact test for categorical variables.

A *p*-value less than 0.05 was considered statistically significant. All analyses were performed using STATA SE version 17.

## Results

**Fig. 1:**
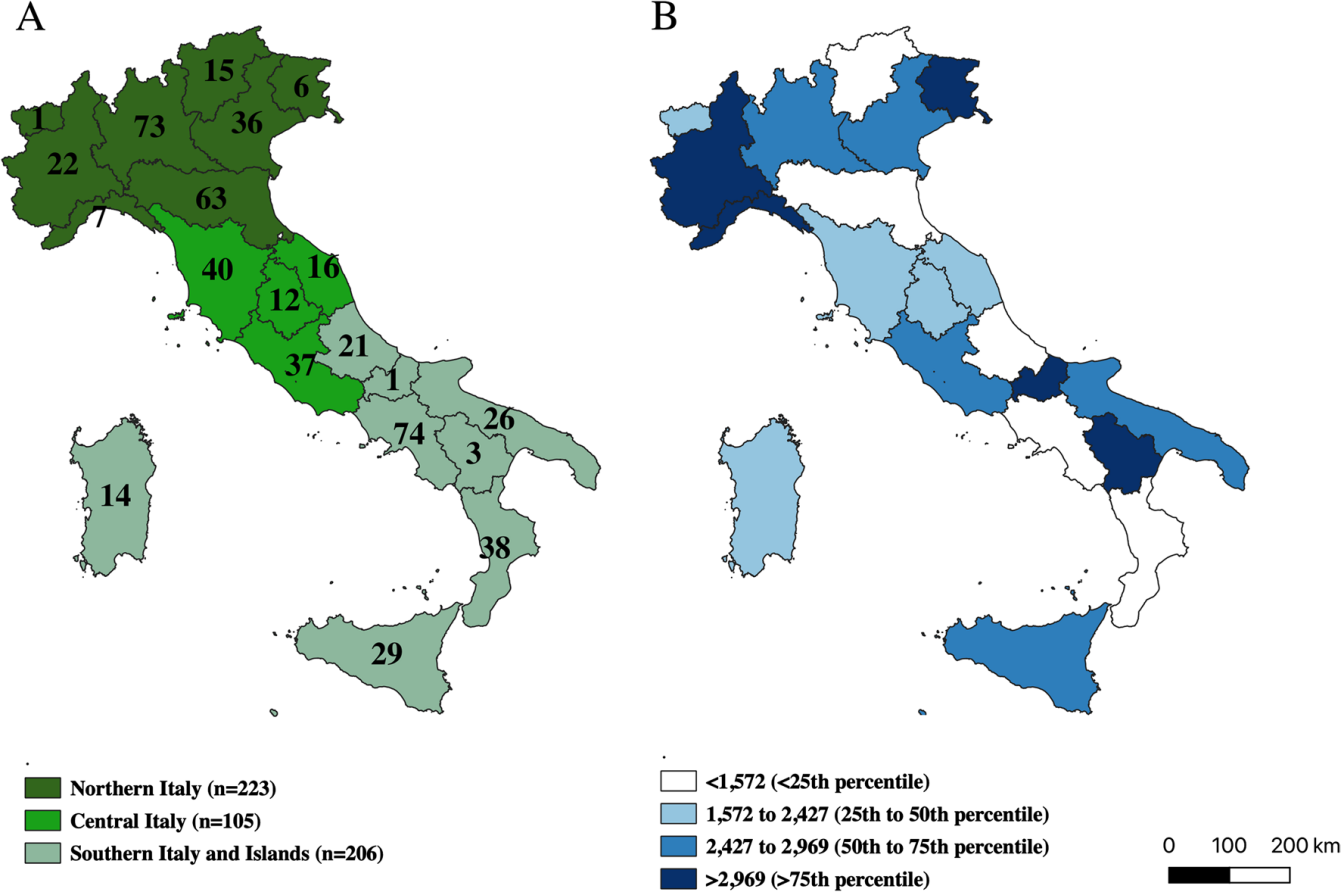
**A** Distribution of Italian CCDDs by Italian region and geographic macro-areas defined according to the Italian National Institute of Statistics (ISTAT) categorization; **B** Estimated mean number of dementia cases per CCDD at the regional level

Overall, 534 CCDDs were identified across Italy, including 223 in the Northern regions, 105 in the Central regions and 206 in the South and Islands (Fig. 1A). The mean number of dementia cases per CCDD was 2065, decreasing from 2405 in the North, 2216 in the Center to 1618 in the South/Islands of Italy (Supplementary table 1). At the regional level, the estimated mean number of dementia cases per CCDD varied widely ranging from 6445 (Molise) to 860 (Calabria) (Fig. 1B, and Supplementary table 1). The profile section of the web-based questionnaire was completed by 511 (96%) out of 534 facilities. The overall response rate was 100% in the North, 97% in the Center, and 91% in South/Islands (Supplementary table1). The data collection form, which collect information on staff, services provided relating to care, non-pharmacological treatments and assistance, type of diagnosis and characteristics of clinical activities based on data referred to 2019, was completed by 450 out of 534 (84%) CCDDs.

CCDDs had a median established duration of 20 years (IQR 9–22), with a similar duration of activity observed across the three different areas of Italy (North 21 (IQR 10–22) years, Center 17 (IQR 5–23), South/Islands 20 (IQR 10–22), *p* = 0.456).

### Main organizational characteristics of CCDDs

A total of 163 branches belonging to 98/511 (19%) CCDDs was identified; each main facility could have from one to five branches (Supplementary table 2).

As regards the setting, territorial and hospital-based CCDDs were the most common types (Table 1). In Northern regions, CCDDs were more frequently placed in hospitals, while territorial facilities were more often available in South/Islands (*p* < 0.001). The median of opening hours was higher in North Italy (*p* < 0.006) and in this area increasing from 12.3 in 2014 to 18 h nowadays; it remained similar in Center and South/Islands (Table 1). More than half (55.1%) of CCDDs were opened 3 or less days in a week, the number of opening days in main facilities was similar according to macro-areas (*p* = 0.081). As regarding branches, no significant difference was found in opening days and hours across the three areas (data not shown).

**Table 1:** Characteristics of Italian CCDDs at the national level and by geographical macro-area, data are expressed as N (%) or median (interquartile range—IQR) and mean (min–max)

*Organizational features Referred to 2022 *(*n* = adherent CCDDs)	Italy (*n* = 511)	North (*n* = 223)	Center (*n* = 102)	South/Islands (*n* = 186)	*p*
*Setting, N* (%)					
Territorial	225 (44.0%)	71 (31.8%)	42 (41.2%)	112 (60.2%)	< 0.001
Hospital	239 (46.8%)	130 (58.3%)	48 (47.1%)	61 (32.8%)	
University	47 (9.2%)	22 (9.9%)	12 (11.8%)	13 (7.0%)	
*Main CCDD opening days/week,*					
median (IQR)	3 (1–5)	4 (2–5)	3 (2–5)	3 (1–5)	0.081
mean (min–max)	3.2 (1–6)	3.3 (1–6)	3.1 (1–6)	3.0 (1–6)	
*Main CCDD opening hours/week,*					
median (IQR)	14 (6–27)	18 (7–31)	13 (7–22)	11 (6–24)	0.006
mean (min–max)	17.6 (1–72)	19.7 (1.5–47)	16.5 (1–63)	15.7 (2–72)	
CCDDs with branches, *N* (%)	98 (19.2%)	45 (20.2%)	21 (20.6%)	32 (17.2%)	0.690
*Referred to 2019 *(*n* = adherent CCDDs operating in 2019)	Italy (*n* = 450)	North (*n* = 202)	Center (*n* = 82)	South/Islands (*n* = 166)	*p*
* Waiting list existence*, *N* (%)	357 (79.3%)	163 (80.7%)	63 (76.8%)	131 (78.9%)	0.756
*Waiting time to access the services, N* (%)					
1–3 months	215 (60.1%)	83 (50.9%)	42 (66.6%)	90 (68.2%)	0.002
4–6 months	85 (23.7%)	52 (31.9%)	14 (22.2%)	19 (14.4%)	
> 6 months	20 (5.6%)	12 (7.4%)	4 (6.4%)	4 (3.0%)	
*Availability of a ICPs (Region, Hospital, Health Local Service, district level)*, *N* (%)	224 (49.8%)	139 (68.8%)	40 (48.8%)	45 (27.1%)	< 0.001
*Staff composition: proportion on the overall staff,* median % (IQR) mean (min–max)					
Neurologists	20% (0–43)	17% (0–33)	27% (0–50)	20% (0–50)	0.015
	26% (0–100)	20% (0–100)	33% (0–100)	29% (0–100)	
Geriatricians	18% (0–46)	22% (0–44)	20% (0–50)	0% (0–40)	0.074
	27% (0–100)	27% (0–100)	34% (0–100)	23% (0–100)	
Psychiatrists	0% (0–0)	0% (0–0)	0% (0–0)	0% (0–0)	< 0.001
	4% (0–100)	3% (0–100)	0.5% (0–14)	7% (0–100)	
Neuropsychologists/psychologists	17% (0–33)	22% (14–33)	9% (0–29)	0% (0–25)	< 0.001
	19% (0–100)	24% (0–83)	17% (0–100)	13% (0–67)	
Other (nurses, social workers, speech therapists, physiotherapists, occupational therapists, cultural mediators, geneticists, interpreters, administrative)	25% (0–40) 25% (0–82)	28% (0–40) 26% (0–75)	0% (0–33) 16% (0–67)	33% (0–50) 27% (0–82)	< 0.001
*Referred to 2020/2021*	Italy (*n* = 450)	North (*n* = 202)	Center (*n* = 82)	South/Islands (*n* = 166)	*p*
*Opening in 2020, N*(%)					
Always open	165 (36.7%)	50 (24.8%)	26 (31.7%)	89 (53.6%)	< 0.001
Closed for < 3 months	133 (29.6%)	73 (36.1%)	31 (37.8%)	29 (17.5%)	
Closed for > = 3 months	124 (27.5%)	63 (31.2%)	20 (24.4%)	41 (24.7%)	
Closed for unknown time	28 (6.2%)	16 (7.9%)	5 (6.1%)	7 (4.2%)	
*Opening in 2021, N (%) *					
Always open	368 (81.8%)	163 (80.7%)	69 (84.2%)	136 (81.9%)	0.594
Closed for < 3 months	39 (8.7%)	21 (10.4%)	5 (6.1%)	13 (7.8%)	
Closed for > = 3 months	32 (7.1%)	11 (5.4%)	7 (8.5%)	14 (8.4%)	
Closed for unknown time	11 (2.4%)	7 (3.5%)	1 (1.2%)	3 (1.8%)	

*ICPs* integrated care pathways

Missing values: waiting list existence (*n* = 23, 5.1%), waiting time to access the services (*n* = 38, 10.6%), staff (*n* = 1, 0.2%), availability of a ICPs (*n* = 39, 8.7%)

Most of structures were coordinated by neurologists (*n* = 236, 46.1%) or by geriatricians (*n* = 215, 42.0%), only 6% were managed by psychiatrists (*n* = 29) and 1.6% by psychologists (*n* = 8). At least two specialized physicians (neurologist, geriatrician, and psychiatrist) were present in 133 (29.5%) facilities, with similar proportion in the macro-areas (North 31%, Center 28%, South/Islands 28%, *p* = 0.790) compared to 20.8% in the previous survey (data not shown) [27],

The total number of healthcare professional employed in the 450 facilities and 163 branches was of 2565 (1270 medical staff, 548 psychologists/neuropsychologists, 447 nurses, 248 other healthcare professionals, 52 administrative staff).

About one-fifth (18%) of total staff were temporary workers, with higher rates among psychologists and neuropsychologists, reaching 34% and 43%, respectively (data not shown). These professionals, on average, accounted for 19% of the staff of a CCDDs with significant differences between macro-areas (Table 1).

Regarding the availability of services during the pandemic, only one-third of facilities were always open in 2020. In 2021, on the other hand, two-thirds of the facilities were open (Table1).

### Services for diagnostic assessment

Table 2 shows the proportions of CCDDs, which provided diagnostic tools directly or through agreement in Italy and in its three different geographical areas. An increase in the availability of all the procedures was observed compared to previous survey [27], from 88 to 94% for neuropsychological assessment, from 43 to 78% for electroencephalogram (EEG), from less than 70 to 81% for brain computer tomography (CT) and magnetic resonance imaging (MRI), cerebrospinal fluid (CSF) biomarkers from 30 to 62%, genetic testing from 27 to 56%, single photon emission computed tomography (SPECT)/positron emission tomography PET FDG from 50 to 77% (data not shown), volumetric and functional MRI from 17 and 23% to over 40% for both (Table 2).

**Table 2:** Diagnostic procedures provided by Italian CCDDs directly or by agreement at the national level and by geographical macro-area, data are expressed as *N* (%) or median (interquartile range—IQR) and mean (min–max)

Diagnostic assessment	Italy (*n* = 450)	North (*n* = 202)	Center (*n* = 82)	South/Islands (*n* = 166)	*p*
*Services provided directly or by agreement, N* (%)					
Clinic assessment	450(100.0%)	202 (100.0%)	82 (100.0%)	166 (100.0%)	1.000
Neuropsychological assessment	423 (94.0%)	197 (97.5%)	75 (91.5%)	151 (91.0%)	0.012
ECG and cardiological examination	382 (84.9%)	176 (87.1%)	65 (79.3%)	141 (84.9%)	0.245
Blood tests	371 (82.4%)	175 (86.6%)	62 (75.6%)	134 (80.7%)	0.066
Brain MRI	366 (81.3%)	171 (84.7%)	65 (79.3%)	130 (78.3%)	0.260
CT Brain scan	366 (81.3%)	169 (83.7%)	63 (76.8%)	134 (80.2%)	0.395
EEG	351 (78.0%)	168 (83.2%)	60 (73.2%)	123 (74.1%)	0.057
PET FDG	335 (74.4%)	165 (81.7%)	57 (69.5%)	113 (68.1%)	0.006
SPECT	314 (69.8%)	148 (73.3%)	51 (62.2%)	115 (69.3%)	0.181
PET amyloid	300 (66.7%)	142 (70.3%)	53 (64.6%)	105 (63.3%)	0.329
Ordinary hospitalization	297 (66.0%)	142 (70.3%)	61 (74.4%)	94 (56.6%)	0.005
CSF markers	281 (62.4%)	153 (75.7%)	47 (57.3%)	81 (48.8%)	< 0.001
Genetic testing	254 (56.4%)	126 (62.4%)	44 (53.7%)	84 (50.6%)	0.065
Day hospital	242 (53.8%)	115 (56.9%)	45 (54.9%)	82 (49.4%)	0.345
Plasma markers	222 (49.3%)	120 (59.4%)	31 (37.8%)	71 (42.8%)	< 0.001
Genetic counseling	218 (48.4%)	106 (52.5%)	34 (41.5%)	78 (47.0%)	0.217
Functional neuroimaging	193 (42.9%)	79 (39.1%)	32 (39.0%)	82 (49.4%)	0.103
Volumetric resonance	182 (40.4%)	74 (36.6%)	32 (39.0%)	76 (45.8%)	0.197
EEG with brain connectivity assessment	150 (33.3%)	61 (30.2%)	23 (28.0%)	66 (39.8%)	0.082
Total number of neuropsychological tests used					
Median (IQR)	23 (12–31)	28 (21–34)	22 (9–31)	14 (8–24)	< 0.001
Mean (min–max)	22 (0–50)	27 (3–50)	21 (0–50)	17 (1–49)	

*ECG* echocardiogram, *MRI* magnetic resonance imaging, *CT* computer tomography, *EEG* electroencephalogram, *PET* *FDG* positron emission tomography fluor  deoxy  glucose, *SPECT* single photon emission computed tomography, *CSF* cerebrospinal fluid

Significant differences were observed in the availability of PET FDG, and CSF biomarkers between CCDDs in the North compared to those in the Center and South/Islands consistent with previous results (*p* = 0.006 and *p* < 0.001, respectively). Also, neuropsychological tests and plasma biomarkers were more frequently used for the diagnostic evaluation in the Northern regions (*p* = 0.012 and* p* < 0.001, respectively) (Table 2).

### Services for care and treatment

The use of telemedicine and the presence of Alzheimer’s Café were available in less than half of facilities, and more frequently in the Northern regions (Table 3). Patients and family counseling services were less available in Central and Southern regions. Cognitive rehabilitation was provided in 67% of CCDDs, with significant differences according to Italian macro-areas. CCDDs collaborated with family associations and third sector organizations in 66% and 50% of structures, with significant lower proportion in Southern regions (*p* < 0.001 and *p* = 0.017, respectively). Structures providing legal aid promotion, legal support, training, and updating activity were less common in the South. On the contrary, home visits and motor tele rehabilitation were offered more frequently in CCDDs in the Center and the South of Italy.

**Table 3:** Care services, psychosocial, educational and rehabilitation treatments and other assistance services provided by Italian CCDDs directly or by agreement at the national level and by geographical macro-area, data are expressed as N (%)

Provision of care services and interventions	Italy (*n* = 450)	North (*n* = 202)	Center (*n* = 82)	South/Islands (*n* = 166)	*p*
*Types of services provided directly or by agreement, N (%)*					
Care					
Planning periodic visits	441 (98.0%)	200 (99.0%)	80 (97.6%)	161 (97.0%)	0.367
Prescribing drug treatment	444 (98.7%)	198 (98.0%)	81 (98.8%)	165 (99.4%)	0.496
Monitoring of drug treatment	445 (98.9%)	199 (98.5%)	81 (98.8%)	165 (99.4%)	0.725
Telemedicine	206 (45.8%)	108 (53.5%)	46 (56.1%)	52 (31.3%)	< 0.001
Use of digital tools for remote monitoring	104 (23.1%)	37 (18.3%)	23 (28.0%)	44 (26.5%)	0.090
Individual patient counseling	363 (80.7%)	177 (87.6%)	64 (78.0%)	122 (73.5%)	0.002
Patient and family counseling	383 (85.1%)	185 (91.6%)	69 (84.1%)	129 (77.7%)	0.001
Individual counseling for family members and caregivers	362 (80.4%)	179 (88.6%)	61 (74.4%)	122 (73.5%)	< 0.001
Information for family and caregivers	393 (87.3%)	188 (93.1%)	69 (84.1%)	136 (81.9%)	0.004
Home visits	250 (55.6%)	98 (48.5%)	45 (54.9%)	107 (64.5%)	0.009
Psychosocial, educational and rehabilitation treatments and interventions					
Cognitive rehabilitation	303 (67.3%)	153 (75.7%)	53 (64.6%)	97 (58.4%)	0.002
Motor rehabilitation	266 (59.1%)	114 (56.4%)	50 (61.0%)	102 (61.4%)	0.580
Speech and language rehabilitation	249 (55.3%)	114 (56.4%)	41 (50.0%)	94 (56.6%)	0.561
Occupational rehabilitation	204 (45.3%)	80 (39.6%)	39 (47.6%)	85 (51.2%)	0.076
Cognitive telerehabilitation	104 (23.1%)	38 (18.8%)	18 (22.0%)	48 (28.9%)	0.070
Motor telerehabilitation	78 (17.3%)	23 (11.4%)	18 (22.0%)	37 (22.3%)	0.011
Digital rehabilitation tools	91 (20.2%)	31 (15.3%)	18 (22.0%)	42 (25.3%)	0.055
Alzheimer’s café	208 (46.2%)	121 (59.9%)	36 (43.9%)	51 (30.7%)	< 0.001
Meeting center	105 (23.3%)	51 (25.2%)	19 (23.2%)	35 (21.1%)	0.643
Mindfulness	69 (15.3%)	20 (9.9%)	18 (22.0%)	31 (18.7%)	0.012
Art therapy	126 (28.0%)	53 (26.2%)	32 (39.0%)	41 (24.7%)	0.046
Sensory stimulation	83 (18.4%)	32 (15.8%)	18 (22.0%)	33 (19.9%)	0.405
Reminiscence therapy	130 (28.9%)	49 (24.3%)	31 (37.8%)	50 (30.1%)	0.067
Reality orientation therapy	153 (34.0%)	61 (30.2%)	35 (42.7%)	57 (34.3%)	0.131
Validation therapy	124 (27.6%)	44 (21.8%)	29 (35.4%)	51 (30.7%)	0.035
Psychotherapy	214 (47.6%)	106 (52.5%)	34 (41.5%)	74 (44.3%)	0.152
Behavioural therapy	183 (40.7%)	79 (39.1%)	35 (42.7%)	69 (41.3%)	0.820
Other assistance services					
Integrated home care	298 (66.2%)	142 (70.3%)	49 (59.8%)	107 (64.5%)	0.196
Day services	285 (63.3%)	141 (69.8%)	61 (74.4%)	83 (50.0%)	< 0.001
Residential service	297 (66.0%)	139 (68.8%)	54 (65.9%)	104 (62.7%)	0.462
Respite hospitalization	265 (58.9%)	138 (68.3%)	51 (62.2%)	76 (45.8%)	< 0.001
Transport service	183 (40.7%)	88 (43.6%)	36 (43.9%)	59 (35.5%)	0.239
Telecare service	110 (24.4%)	56 (27.7%)	20 (24.4%)	34 (20.5%)	0.274
Telephone listening points	206 (45.8%)	107 (53.0%)	34 (41.5%)	65 (39.2%)	0.021
Legal aid promotion	242 (53.8%)	126 (62.4%)	47 (57.3%)	69 (41.6%)	< 0.001
Legal support	250 (55.6%)	129 (63.9%)	54 (65.9%)	67 (40.4%)	< 0.001
Clinical-epidemiological research activities	214 (47.6%)	104 (51.5%)	41 (50.0%)	69 (41.6%)	0.147
Training and professional updating activities	296 (65.8%)	147 (72.8%)	54 (65.9%)	95 (57.2%)	0.008
Secondary prevention activities on MCI Patients	272 (60.4%)	126 (62.4%)	50 (61.0%)	96 (57.8%)	0.671
Contacts with family associations	297 (66.0%)	151 (74.8%)	58 (70.7%)	88 (53.0%)	< 0.001
Contacts with third sector organizations	225 (50.0%)	114 (56.4%)	42 (51.2%)	69 (41.6%)	0.017

*MCI* mild cognitive impairment

### Diagnosis of patients followed by CCDDs and Clinical activities of CCDDs

The most frequent diagnosis was dementia (60%) followed by mild cognitive impairment (20%) and subjective memory disorder (10%), the latter was less frequent in patients followed in the North (Table 4). Among dementia diagnoses, Alzheimer was prevalent, followed by mixed and vascular dementia, which were significantly more frequent in the South/Islands (*p* < 0.001 for both) (Table 4).

**Table 4:** Diagnosis and characteristics of clinical activities of Italian CCDDs at the national level and by geographical macro-area, data are expressed as N (%) or median (interquartile range—IQR) and mean (min–max)

Diagnosis and characteristics of clinical activities of CCDDs	Italy (*n* = 450)	North (*n* = 202)	Center (*n* = 82)	South/Islands (*n* = 166)	*p*
*Type of diagnosis* Median% (IQR) Mean% (min–max)					
Subjective memory disorder	10% (5–15)	5% (4–10)	10% (5–15)	10% (5–20)	< 0.001
	12% (0–62)	9% (0–41)	12% (0–50)	15% (0–62)	
Mild cognitive impairment	20% (15–30)	20% (15–27)	20% (15–30)	20% (15–28)	0.526
	21% (0–80)	21% (1–50)	22% (5–60)	21% (0–80)	
Dementia	60% (50–70)	62% (50–75)	60% (50–70)	60% (45–70)	0.065
	59% (10–100)	62% (13–95)	58% (20–95)	56% (10–100)	
Other	5% (1–10)	5% (1–10)	5% (1–10)	5% (1–10)	0.569
	8% (0–49)	8% (0–44)	7% (0–49)	8% (0–40)	
*Type of dementia* Median% (IQR) Mean % (min–max)					
Alzheimer’s disease	40% (30–50)	40% (32–50)	50% (40–60)	40% (22–50)	< 0.001
	41% (5–83)	43% (9–83)	48% (5–80)	36% (5–80)	
Frontotemporal dementia	5% (4–10)	5% (5–10)	5% (3–10)	5% (5–10)	0.230
	7% (0–40)	7% (0–30)	6% (0–22)	8% (0–40)	
Vascular dementia	15% (10–20)	15% (10–20)	15% (10–20)	20% (10–28)	< 0.001
	17% (0–70)	15% (0–35)	16% (0–40)	21% (1–70)	
Lewy’s bodies dementia	5% (3–10)	5% (4–10)	5% (4–10)	5% (2–8)	< 0.001
	7% (0–34)	8% (0–34)	7% (0–20)	5% (0–30)	
Mixed dementia	20% (15–30)	20% (12–30)	20% (10–25)	25% (15–35)	< 0.001
	23% (0–70)	21% (0–60)	20% (3–70)	26% (0–70)	
Other	3.5% (1–5)	4% (1–5)	2% (0–5)	3% (0–5)	0.146
	5% (0–60)	6% (0–60)	3% (0–10)	4% (0–40)	
Number of patients in charge annually visited (assessed at least one time per year)					
Median (IQR)	505(282–973)	600(314–1200)	600(305–875)	400(245–800)	0.003
Mean (min–max)	791(24–5000)	893(80–4830)	813(100–4500)	660(24–5000)	
Average of patients assessed per month,					
Median (IQR)	64 (35–120)	78 (40–149)	80 (35–104)	50 (30–90)	0.003
Mean (min–max)	95 (4–600)	110 (7–600)	92 (10–380)	78 (4–450)	
Average of patients assessed for the first time per month					
Median (IQR)	20 (10–37)	22 (10–38)	20 (12–50)	19 (12–30)	0.485
Mean (min–max)	30 (0–200)	32 (3–200)	32 (3–110)	27 (2–170)	
Percentage of patients that received psychosocial, educational and rehabilitation treatment in the last year					
Median% (IQR)	25% (0–100)	22% (0–93)	29% (0–100)	28% (0–100)	0.587
Mean% (min–max)	15% (5–31)	15% (6–30)	20% (5–50)	15% (5–40)	
Percentage of carers (expressed as average number of patients and carers) that received psychosocial and educational support after dementia diagnosis in the last year					
Median% (IQR)	24% (0–100)	24% (0–100)	26% (0–100)	25% (0–100)	0.391
Mean% (min–max)	15% (5–35)	15% (10–30)	15% (5–30)	13% (0–40)	
Percentage of patients with dementia that received an antipsychotics prescription in the last year					
Median% (IQR)	36% (0–100)	31% (1–85)	38% (3–80)	41% (0–100)	< 0.001
Mean% (min–max)	30% (20–50)	30% (15–40)	35% (25–50)	40% (22–60)	

Missing values: type of diagnosis (*n* = 102, 22.7%), type of dementia (*n* = 118, 26.2%), number of patients in charge annually visited (*n* = 82, 18.2%)*,* average of patients assessed per month (*n* = 26, 5.8%), average of patients assessed for the first time per month (n = 29, 6.4%), average time spent per patient at first or control visit (*n* = 9, 2.0%), percentage of patients that received a complete neuropsychological assessment in the last year (*n* = 125, 27.8%), percentage of patients with dementia that received non-pharmacological support in the last year (*n* = 193, 42.9%), percentage of carers (expressed as average number of patients and carers) that received non-pharmacological support after dementia diagnosis in the last year (*n* = 199, 44.2%), percentage of patients with dementia that received an antipsychotics prescription in the last year (*n* = 130, 28.9%)

The median number of patients annually under the care of CCDDs and followed monthly was 505 (IQR 282–973) and 64 (IQR 35–120), respectively, with significant geographical differences (Table 4). The percentage of patients with dementia receiving a complete neuropsychological assessment is about 60% and decreased of 20 points respect to 2016 [27]. The proportion was significantly higher in the South of Italy where a lower median number of neuropsychological tests was found compared to the North and Center (Table 2). On the other hand, the number of patients and caregivers that received psychosocial, educational, and rehabilitation treatment and support increased from 10% in the previous survey to about 25% for both [27].

Patients with dementia receiving antipsychotics rose from 30% in 2014 to 36%; this proportion was higher in the center and South/Islands compared to the North [27].

## Discussion

This survey provided an up-to-date overview of the current clinical situation of CCDDs in Italy. Similar to the previous survey [27], our study included a substantial large number of respondents (96%), as a result of an extensive interaction with regional representatives, and confirmed the wide heterogeneity of organizational aspects (e.g., staffing, number of patients, waiting times, assessment, and services provided) among CCDDs in three geographical macro-areas of Italy.

The number of CCDDs has decreased since the previous survey (534 compared to 597). However, it is important to note that there are 163 branches, indicating a greater presence of dispersed services. Unfortunately, once again, the distribution of CCDDs does not align with the epidemiological estimates of the number of patients with dementia (Fig. 1B, Supplementary table 1). Significant disparities are observed within the same geographical macro-areas, such as Liguria and Piemonte compared to Emilia Romagna in Northern Italy. It appears that the CCDDs in Italy have developed without planning the provision of services based on the epidemiological frequency of the disease, i.e., linking the development of new services to geographical areas known to have a high prevalence of dementia. Similar deficiencies in the planning of memory clinics were found in the Irish survey [12].

Although more than half of the CCDDs are still open 3 days a week or less, there has been an increase in the number of weekly opening hours over the last 5 years, this corresponds to an increase in the number of patients evaluated per year and per month. As a result, the overall number of patients seen in one year in the CCDDs increased by 10%, with a notable increase in the South (60%) between 2014 and 2019. This characteristic was also observed in a study conducted in the UK, albeit at a higher rate [30].

While it is challenging to compare the organization of different healthcare systems, the frequency of multidisciplinary teams with specialized physicians (neurologist, geriatrician, and psychiatrist) observed in CCDDs (29.5%) is the highest compared to other countries [12, 13].

International guidelines [31] strongly recommend the use of neuropsychological assessment in the diagnosis of dementia, and the increased use of psychologists with neuropsychological training is an encouraging sign.

Unfortunately, only a third of CCDDs have a neuropsychologist present, and a large proportion of these professionals still need to be employed full-time (data not shown). However, several studies have shown that multidisciplinary teams are able to provide appropriate diagnostic assessment and management for neurocognitive disorders [18, 32].

The impact of the COVID-19 pandemic in Italy has been dramatic, especially for people with dementia and their caregivers [33–35]. CCDDs are the core service for people with dementia and their caregivers, and due to preventive restrictions, there was a partial closure of the service in 2020 and 2021 affecting many facilities, especially in Northern Italy. This closure is in line with epidemiological data on the spread of the virus.

New pharmacological therapies with monoclonal antibodies require an extensive advanced medical infrastructure for safe administration [36]. This infrastructure includes increased access to MRI and PET facilities, laboratory facilities to analyze CSF for Alzheimer’s disease biomarkers and testing blood APOE [37]. In this survey, data on the diagnostic tools used showed an increase in the utilization of CSF, neuroimaging and PET, with marked heterogeneity among different geographical areas and with implications for the possible approval of a disease-modifying therapy for dementia (Table 2). With disease-modifying drugs on the horizon, early detection of mild cognitive impairment is a critical step. About 1 in 5 patients evaluated at CCDDs in Italy receive a diagnosis of MCI. Compared to the 2014 survey, there has been a 10% decrease in dementia diagnoses and an increase in subjective memory disorders (5% vs. 12%) [27]. A decreased of dementia diagnosis was also observed in the Netherlands [13]. The distribution of diagnoses in our study aligns with previously published registries. In particular, the proportions of AD and VaD patients in the Swedish National Dementia Registry were quite similar [38]. In the South and the Islands there is a significant variation in subtype diagnosis, which could be explained by differences in risk factors across different geographical areas. This variation could be related to the significant difference in the number of neuropsychological tests used in the three macro-areas, which is significantly lower in Southern Italy and the Islands (Table 2). Moreover, the greater prevalence of territorial CCDDs in this part of the country, to the detriment of university-type CCDDs, could justify a less comprehensive diagnostic process.

The Lancet commission recommendations for post diagnosis care in 2020 [6] include taking care of physical and mental health, social care and support, specific multicomponent interventions to decrease neuropsychiatric symptoms and specific interventions for family careers. In addition, numerous studies have shown that proactive management of Alzheimer’s and other dementias can improve the quality of life of people with these disorders and their caregivers [18]. Regarding these aspects, while all CCDDs provide post-diagnosis drug treatment and drug treatment monitoring, there is variation in the availability of psychosocial and rehabilitative interventions, with less than half of facilities offering occupational therapy and two-thirds offering cognitive rehabilitation. The survey clearly indicates that in 2019, a significant number of patients and caregivers were not provided with psychosocial and educational treatment, or rehabilitation for patients alone.

Compared to the previous survey, information was collected on the use of digital tools and telemedicine services for remote patient monitoring, which were used by about a quarter and half of the CCDDs, respectively. The focus on digital services has significantly increased with the impact of the pandemic [39], and the data collected in this survey for 2019 shows that the system needs to be prepared to deal with the demands of social distancing.

Care for chronic conditions needs integration across settings and providers, and continuity of care (from first point of contact with the patient) [40]. The implementation of an “integrated care pathways” (ICPs), can be a crucial element in achieving the optimal management of people with complex chronic disorders, such as dementia [41] and the CCDDs have a pivotal role in the management of dementia and cognitive disturbances throughout the natural history of the disease. The goals of the Italian NDP include the establishment of an integrated dementia network and the implementation of integrated management [23, 42] across all Italian regions. This survey showed that only 50% of the CCDDs had a referring integrated care pathway (ICP) for people with dementia (Table 1). The lack of available territorial ICPs does not correspond to the requirements stated in the NDP.

A limitation of this study is the use of self-reported information, which can be influenced by subjective interpretations that may not correspond perfectly to the actual data. However, a comparison with data from previous surveys, the reports from other countries and with the feedback received from delegates in the different regions demonstrates a certain level of reliability in the results, which aim to provide an overview of the Italian situation. Due to the pandemic-related shutdown periods in 2020–2021, it was necessary to collect information related to 2019, which was the last year before 2022 with complete operations. It is possible that the pandemic significantly had a significant impact on the provision of some services, such as remote monitoring and telemedicine. However, although updating and monitoring activities were carried out throughout the survey period, it is not possible to guarantee that all data presented in this report relate to 2019.

One of the strengths of this survey is the remarkable response rate. The active cooperation of the regional delegates has made it possible to obtain official lists of CCDDs in the area and cooperate on the practical use of this information. The survey has revealed previously unexplored phenomena, such as the significant presence of CCDD branches throughout the country.

A continuous update of services, their organizational structure, patient numbers, and medical and social care aspects can be used to produce activity reports. It can also lead to research hypotheses and improvements in patient care [43].

To achieve this goal, it would be important to set up a national dementia information system, as it has been implemented in other countries [43] and envisioned by the Italian NDP. Planning the development of CCDDs in areas with higher estimates of people with dementia or MCI is crucial. Therefore, it may be useful to utilize health information systems to identify people with MCI or dementia to develop new services in underserved areas [44]. Currently, many Italian regions are making efforts to create a computerized medical record for CCDDs [25] to be included in a future national information system.

The results of this survey will allow an update of all structural and human resources highlighting the disparities between different Regions and territories in the field of prevention, diagnosis, management, and pharmacological, cognitive, and psychosocial treatment. Furthermore, these results will contribute to the development of national guidelines and care standards for CCDDs.

In a heterogeneous Italian and international context for memory clinics some recommendations for the deployment of second-generation memory clinics [45, 46] have been proposed and a reorganization of the CCDDs in Italy into three levels of increasing complexity was suggested [47].

However, the main conclusion that can be drawn from comparing this survey with one conducted in 2014, is that there is an ongoing, albeit slow, process of strengthening the network of services in Italy. There are still many challenges to be faced and overcome which, however, require a solid economic investment in public health [48].

### Supplementary Information

Below is the link to the electronic supplementary material.

Supplementary file1 (DOCX 31.3 KB)

## Data Availability

Data will be available  upon request.
