# Supplementary material for: A new national survey of centers for cognitive disorders and dementias in Italy
Source: Neurol Sci. 2023 Aug 18;45(2):525–38. doi: 10.1007/s10072-023-06958-8 (PMC10791890; doi:10.1007/s10072-023-06958-8)
Supplement: Supplementary file 1 — Supplementary file1 (DOCX 31.3 KB) [file 10072_2023_6958_MOESM1_ESM.docx]

Ilaria Bacigalupo^1^, Francesco Giaquinto^2^, Emanuela Salvi^3^, Giulia Carnevale^4^ , Roberta Vaccaro^4,5^, Fabio Matascioli^4,6^, Giulia Remoli^7^, Nicola Vanacore^1^, Patrizia Lorenzini^1^ and the Permanent Table of the National Dementia Plan Study Group and the CCDD Study Group

^1^National Centre for Disease Prevention and Health Promotion, Italian National Institute of Health, Rome, Italy,

^2^ Department of Human and Social Sciences, University of Salento, Lecce, Italy,

^3^ National Center for Drug Research and Evaluation, Italian National Institute of Health, Rome, Italy,

^4^Italian National Institute of Health FONDEM Study Group

^5^GINCO, Aware Aging Group, Como, Italy,

^6^ TAM Onlus, Social Cooperative, Naples, Italy,

^7^ Neurology Section, University of Milano-Bicocca, Milan, Italy

Corresponding author: Ilaria Bacigalupo

Submitted to Neurological Sciences

**Supplementary tab. 1** Distribution of Italian CCDDs according to regions and macro-areas, estimated number of dementia and estimated number of cases per CCDDs, response rate for the profile section and for the data collection form

| **Regions** | **Estimated cases of dementia*** | **N of CDCD** | **Ratio cases/CDCD** | **N of CDCD filling profile section** | **N of CDCD filling data collection form and in activity in 2019** |
| --- | --- | --- | --- | --- | --- |
| Emilia Romagna | 90250 | 63 | 1433 | 63 (100%) | 60 (95%) |
| Friuli Venezia Giulia | 26121 | 6 | 4354 | 6 (100%) | 6 (100%) |
| Liguria | 38202 | 7 | 5457 | 7 (100%) | 6 (86%) |
| Lombardia | 180896 | 73 | 2478 | 73 (100%) | 65 (89%) |
| Piemonte | 90271 | 22 | 4103 | 22 (100%) | 21 (95%) |
| Trentino Alto Adige | 18035 | 15 | 1202 | 15 (100%) | 13 (87%) |
| Valle d'Aosta | 2375 | 1 | 2375 | 1 (100%) | 0 (0%) |
| Veneto | 90234 | 36 | 2507 | 36 (100%) | 31 (86%) |
| **North** | **536384** | **223** | **2405** | **223 (100%)** | **202 (91%)** |
| Lazio | 101195 | 37^§^ | 2735 | 34 (92%) | 28 (76%) |
| Marche | 32458 | 16 | 2029 | 16 (100%) | 15 (94%) |
| Toscana | 79646 | 40 | 1991 | 40 (100%) | 30 (75%) |
| Umbria | 19420 | 12 | 1618 | 12 (100%) | 9 (75%) |
| **Centre** | **232719** | **105** | **2216** | **102 (97%)** | **82 (78%)** |
| Abruzzo | 25778 | 21 | 1228 | 20 (95%) | 16 (76%) |
| Basilicata | 10661 | 3 | 3554 | 3 (100%) | 2 (67%) |
| Calabria | 32666 | 38 | 860 | 34 (89%) | 32 (84%) |
| Campania | 78551 | 74 | 1062 | 73 (99%) | 68 (92%) |
| Molise | 6445 | 1 | 6445 | 1 (100%) | 1 (100%) |
| Puglia | 68451 | 26 | 2633 | 17 (65%) | 12 (46%) |
| Sardegna | 30364 | 14 | 2169 | 14 (100%) | 12 (86%) |
| Sicilia | 80453 | 29 | 2774 | 24 (83%) | 23 (79%) |
| **South/Islands** | **333369** | **206** | **1618** | **186 (91%)** | **166 (81%)** |
| **Total** | **1102472** | **534** | **2065** | **511 (96%)** | **450 (84%)** |

*****estimated cases were calculated by applying European prevalence rates (Bacigalupo et al. JAD 2018) to the ISTAT 2022 over-65 population divided by region

**Supplementary tab. 2** Distribution of Italian CCDDs according to presence or absence of branches and according to number of branches per regions and macro-area

| **Regions** | **Facilities without branches** | **Facilities with at least 1 branches** | **Facilities with 1 branch** | **Facilities with 2 branches** | **Facilities with 3 branches** | **Facilities with 4 branches** | **Facilities with 5 branches** | **Total of branches** |
| --- | --- | --- | --- | --- | --- | --- | --- | --- |
| Emilia Romagna | 55 | 8 | 4 | 3 | 1 |  |  | 13 |
| Friuli Venezia Giulia | 5 | 1 | 1 |  |  |  |  | 1 |
| Liguria | 1 | 6 | 1 | 2 | 2 | 1 |  | 15 |
| Lombardia | 66 | 7 | 6 |  | 1 |  |  | 9 |
| Piemonte | 14 | 8 | 3 | 4 | 1 |  |  | 14 |
| Trentino Alto Adige | 12 | 5 | 4 | 1 |  |  |  | 6 |
| Valle d’Aosta | 1 | 0 |  |  |  |  |  | 0 |
| Veneto | 26 | 10 | 5 | 2 | 3 |  |  | 18 |
| **North** | **180** | **45** | **24** | **12** | **8** | **1** | **0** | **76** |
| Lazio | 27 | 7 | 5 |  |  | 1 | 1 | 14 |
| Marche | 11 | 5 | 4 | 1 |  |  |  | 6 |
| Toscana | 33 | 7 | 2 | 4 | 1 |  |  | 13 |
| Umbria | 10 | 2 |  | 2 |  |  |  | 4 |
| **Centre** | **81** | **21** | **11** | **7** | **1** | **1** | **1** | **37** |
| Abruzzo | 19 | 1 | 1 |  |  |  |  | 1 |
| Basilicata | 3 | 0 |  |  |  |  |  | 0 |
| Calabria | 32 | 2 | 2 |  |  |  |  | 2 |
| Campania | 56 | 17 | 14 | 2 |  |  | 1 | 23 |
| Molise | 1 | 0 |  |  |  |  |  | 0 |
| Puglia | 13 | 4 | 4 |  |  |  |  | 4 |
| Sardegna | 11 | 3 | 1 |  |  | 2 |  | 9 |
| Sicilia | 19 | 5 | 3 |  | 1 |  | 1 | 11 |
| **South/Islands** | **154** | **32** | **25** | **2** | **1** | **2** | **2** | **50** |
| **Total** | **413 (80.8%)** | **98 (19.2%)** | **60 (11.7%)** | **21 (4.1%)** | **10 (1.9%)** | **4 (0.8%)** | **3 (0.6%)** | **163** |
